# Development of a Portable Electronic Nose System for the Detection and Classification of Fruity Odors

**DOI:** 10.3390/s101009179

**Published:** 2010-10-15

**Authors:** Kea-Tiong Tang, Shih-Wen Chiu, Chih-Heng Pan, Hung-Yi Hsieh, Yao-Sheng Liang, Ssu-Chieh Liu

**Affiliations:** Department of Electrical Engineering, National Tsing Hua University/No. 101, Sec. 2, Kuang-Fu Road, Hsinchu, Taiwan; E-Mails: marscloud@hotmail.com (S.W.C.); u931814@oz.nthu.edu.tw (C.H.P.); u941810@oz.nthu.edu.tw (H.Y.H.); u931837@oz.nthu.edu.tw (Y.S.L.); u931427@oz.nthu.edu.tw (S.C.L.)

**Keywords:** portable electronic nose system, sensor array, E-Nose, fruity odor detection

## Abstract

In this study, we have developed a prototype of a portable electronic nose (E-Nose) comprising a sensor array of eight commercially available sensors, a data acquisition interface PCB, and a microprocessor. Verification software was developed to verify system functions. Experimental results indicate that the proposed system prototype is able to identify the fragrance of three fruits, namely lemon, banana, and litchi.

## 1. Introduction

Olfaction is one’s sense of smell and a primary human sensory system. The detection of odors has been applied to many industrial applications, including indoor air quality [1,2], health care, safety and security, environmental monitoring [3–5], quality control of food products [6–8], medical diagnosis [9–12], psychoanalysis [13], agriculture [14], pharmaceuticals, military applications, and detection of hazardous gases, to name but a few. The biological nose is an obvious choice for such applications, but there are some disadvantages to having human beings perform these tasks due to a variety of reasons such as fatigue, infections, mental state, subjectivity, exposure to hazardous materials, individual variables, *etc.*, and generally it is economically unfeasible to invest a large amount of money in training for tasks that last a relatively short time.

Research into alternative olfactorial sensing methods has come a long way since its introduction in 1982 [15]. Over the past 40 years, there have been numerous attempts to build instruments that function as an electronic nose [16]. Although several commercial E-Nose products are available on the market, many of them are bulky, and require a desktop or laptop computer, which makes them unsuitable for portable applications. There are indeed a number of modern small electronic noses, such as the “Diagnose” from C-it of the Netherlands (11 × 18 × 7 cm) and the Artinose from SYSCA AG Germany (17 × 26 × 14 cm), but they are still too expensive for widespread adoption. In addition, existing electronic noses are still unable to perform particularly well because the most commonly used sensors are inadequate for the discriminating tasks required of them. As such, E-Nose products are still difficult to commercialize and the quest for a small, lightweight, and inexpensive E-Nose system has continued in recent years [17–25]. One of the reasons for the difficulty in reducing the size of E-Nose systems is the need to perform odor signal manipulation and classification, which demand high powered central processing units (CPUs), due to the complexity of the algorithms involved. However, many E-Nose applications may only be required to perform relatively simple tasks, and thus, do not require such complicated algorithms. For these kinds of applications, a microprocessor could replace the CPU, making the concept of a portable E-Nose system feasible.

In this paper, we report on the development of a portable E-Nose system prototype using an 8051 microprocessor embedded with a K-nearest-neighbor (KNN) algorithm for odor classification. We have verified the functionality and accuracy of the device with a program we developed, written in LabVIEW. The E-Nose system has successfully detected and classified the odor of three fruits, namely, lemon, banana, and litchi.

## 2. The Proposed E-Nose System

**Figure 1.**
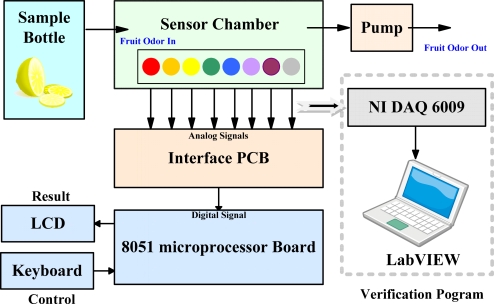
Block diagram of the proposed E-Nose system.

Figure 1 shows a block diagram of the proposed E-Nose system, comprising a sensor array, an interface printed circuit board (PCB), and an 8051 microprocessor board embedded with a pattern recognition algorithm, as well as a verification program. Sensor responses pass through a data acquisition card (DAQ) to a laptop with a self-developed LabVIEW program for the purpose of verifying the function of the portable E-Nose system.

### 2.1. Sensors

One approach to developing a chemical sensing system is to mimic mammalian olfaction. Over 1,000 different receptor genes have been identified in the olfactory system of mammals. Learning from the mammalian system, an array of different sensors is used for odor identification, with each sensor designated to respond to a number of different chemicals. In such an array, no individual sensor responds solely to a specific odor. Rather, the collective response of the entire array produces a unique pattern for the odor of interest. Ideally, to respond to the largest cross-section of analytes, the elements of the sensor array have to possess as much chemical diversity as possible. Within the range of this diversity, the sensor array produces a distinct pattern, taken as an odor signature (odor fingerprint), that can be utilized for odor classification and identification.

This operational principle has the advantage of being able to identify and classify a complex mixture of odors, such as those of fruits, over a one-to-one sensing mode (each sensor responds to a specific odor). In practical applications, odors of interest are usually complex mixtures, rather than pure gases [26]. The fragrance of a fruit, for example, is a complex combination of dozens of individual scents [27–30]. This complexity makes it almost impossible to find sensors corresponding to every individual component of gas mixture. For instance, banana aroma comprises several ester groups, and litchi contains higher amounts of monoterpene hydrocarbons in its scent. The odor thresholds of the human nose to these gaseous constituents generally fall in the range of ppb [31–33]. However, a number of researchers have shown that an electronic nose could classify fruit very nearly as well as a panel of tasters [34]. In this manner, an E-Nose could be useful for the classification of the odor of fruits.

Table 1 lists the eight commercial FIGARO^®^ sensors that form the sensor array. The typical sensing material of the FIGARO^®^ TGS gas sensors is tin oxide (SnO_2_). When SnO_2_ is heated to a specific temperature in the air, oxygen is adsorbed and electrons accumulate on the crystalline surface. These electrons are transferred to the absorbed oxygen, resulting in a positive charge remaining within a space charged layer. As a result, surface potential is created, which serves as a potential barrier to the free exchange of electrons, which would result in a change in resistance. In the presence of deoxidizing gas, the density of the negatively-charged oxygen at the surface would decrease, thus lowering the barrier height and resistance [35–38]. Three sets of identical sensors were incorporated (TGS822, TGS825, and TGS826) in the sensor array for the following reasons:

1. To increase the effectiveness of the sensor: For example, if TGS822 responds to a specific odor, two responses could be recorded, due to the presence of two of the same kind of sensors.
2. To investigate the behavior of identical sensors: Sensors of the same kind may not necessarily behave in exactly the same way. This behavior was investigated during the experiment.
3. In the future, algorithms will be incorporated to average the signals among identical sensors to tune out background noise and interference from temperature or humidity.

### 2.2. Interface PCB

**Figure 2.**
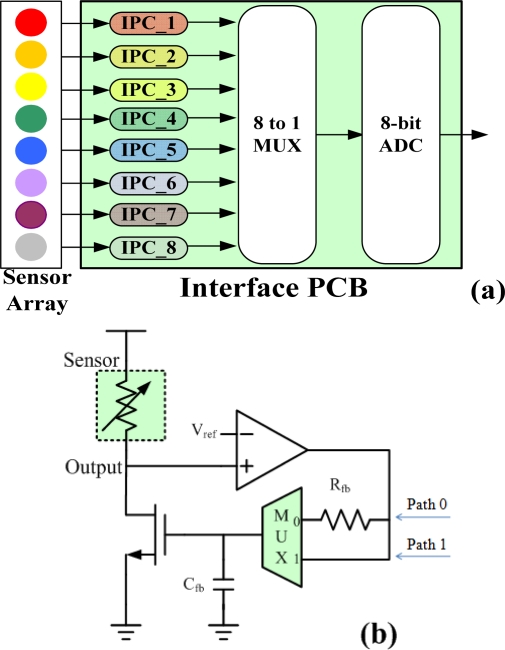
**(a)** Block diagram of the interface PCB; **(b)** Basic architecture of the IPC.

Because the array consists of eight sensors, the interface PCB includes eight interface processing circuits (IPC), an eight to one multiplexer (MUX), and an 8-bit analog-to-digital converter (ADC). The eight interface processing circuits are connected to the eight sensors, which actively adapt the circuit to a preset baseline voltage. The multiplexer reduces the need for multiple ADCs by scanning the eight channels and choosing one channel at a time. The ADC converts sensor data into a digital form for data processing. Figure 2(a) shows a block diagram of the interface PCB. Figure 2(b) shows the basic architecture of the interface processing circuit (IPC), which operates in one of the two following modes:

1. Adaptation mode: in this mode, the circuit adjusts its operating point to a preset baseline voltage. The multiplexer chooses path “1” in Figure 2(b), to equalize the output voltage with the reference voltage V_ref_, which is set as the baseline value prior to sensing odors. In this mode, the NMOS transistor operates as a variable current source. At the end of the adaptation mode, the circuit enters the sensing mode, the gate voltage of the transistor becomes stable, and the transistor operates as a constant current source. After completing the adaptation mode, the E-Nose system is ready to accept input gas.
2. Sensing mode: in this mode, the circuit is ready for sensing. The multiplexer chooses path “0” in Figure 2(b), to form a negative feedback loop, which establishes the gate voltage of the NMOS. Due to a large time constant R_fb_C_fb_, the gate voltage of the NMOS can be maintained a long enough time, comparing with the sensor response time. As a result, the IPC responds to the sensor while tuning out background signals; which is similar to the process performed by biological noses. In this mode, variations in the sensor resistance are translated to a change in output voltage, which is fed into an ADC through an eight to one MUX, whereupon, the ADC output is send to the 8051 microprocessor.

### 2.3. 8051 Microprocessor

The 8051 microprocessor was chosen from the many available, for two reasons:

1. The ability to perform mathematical calculations, *i.e.*, it can perform algorithms to a certain extent, provided the algorithms are not too complicated.
2. The availability of open source code. Because the 8051 microprocessor is available as an open source module, as long as its capability of handling necessary signal processing and process classification algorithms, can be verified, it can be integrated in a future system-on-chip (SoC) design.

After receiving the signal from the ADC, the 8051 microprocessor processes the sensor data. Before gas enters the system, the 8051 microprocessor reads the sensor resistance as its baseline resistance R_b_. When the gas flows into the chamber, the 8051 determines the steady-state value of the sensor resistance R_sense_, and calculates the percentage ratio of resistance change (R_sense_ − R_b_)/R_b_. The collective resistance change ratios of the eight sensors form a pattern according to the input odor, and the 8051 takes this odor pattern into one of its two operational modes, namely, training mode or testing mode described in Section 4. A KNN algorithm is embedded in the 8051 to perform odor classification.

## 3. Sensor Data Acquisition and Odor Classification Interface

**Figure 3.**
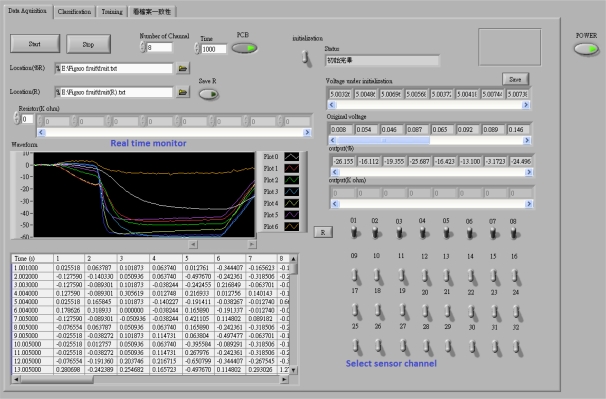
The data acquisition interface.

Running parallel to the 8051 microprocessor, sensor data enters a laptop computer through a National Instrument data acquisition card (interface card: NI DAQ 6009), with a LabVIEW program developed for this study, to characterize sensor and odor data and verify possible classification algorithms. Three data processing interfaces were developed to operate the E-Nose system. These include a data acquisition interface, a training interface, and a classification interface. Figure 3 shows a screenshot of the operating window of the program.

The data acquisition interface records changes in sensor resistance, and plots the change ratio of sensor resistance ΔR/R (ΔR = R_sense_ − R_b_) in real-time. The recorded data builds pattern recognition models for performing classification in the other two interfaces. The training interface uses data stored by the data acquisition interface, to build a classification model, which is used to recognize odors in the classification interface. A radar plot of the odor is shown by the interface for the user to observe.

Users can read and classify odors through the classification interface, which implements six different algorithms, including nearest neighbor (NN), K-nearest neighbor (KNN), support vector machine (SVM), principle component analysis with nearest neighbor (PNN), principle component analysis with K-nearest neighbor (PKNN), and principle component analysis with support vector machine (PSVM). Performing six different algorithms simultaneously enables the user to investigate and compare the efficiency and accuracy among each of the algorithms. The classification results, the “smell print”, and PCA plots are also shown on the interface.

## 4. Experimental Results and Discussion

**Figure 4.**
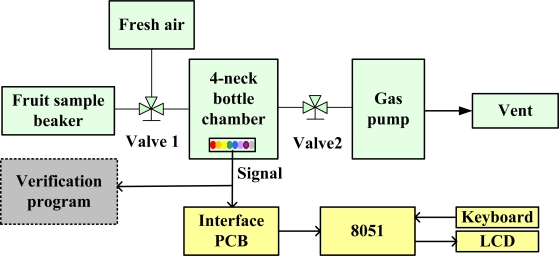
The gas testing setup for the proposed E-Nose system.

Figure 4 shows the setup for the gas testing component of the proposed E-Nose system, which comprises a FIGARO^®^ sensor array, an interface PCB, an 8051 microprocessor board with keyboard and an LCD monitor, a fruit sample beaker, a gas pump, a 4-neck bottle chamber, and the verification program (a NI 6009 DAQ card and a PC with self-developed LabVIEW program). The FIGARO^®^ sensor array is placed in the 4-neck bottle chamber. One of the tubes of the 4-neck bottle chamber is the input pathway, which is controlled by the three phase Valve1, to sample fruit odors or fresh air. Another tube, controlled by Valve2, connects the 4-neck bottle chamber to the gas pump. A signal line connects the sensor array to the interface PCB and the program to verify the feasibility of the system. The interface PCB outputs the data to the 8051 microprocessor for further data processing. A keyboard controls the operation of the 8051 microprocessor, and the results are shown on the LCD.

### 4.1. Operating Procedure

The proposed E-Nose system was tested with the odors of three fruits, namely lemon, banana, and litchi. The odors were prepared by placing samples of fruit in beakers sealed with a membrane. The operational procedures were as follows:

1. The 8051 was set to testing/training mode.
2. Valve1 was closed, and Valve2 opened. The vacuum pump was turned on for 20 seconds to pump the gas out of the 4-neck bottle chamber.
3. Valve1 was opened to connect the 4-neck chamber to the fruit sample beaker. Valve2 was closed, and the vacuum pump was turned off for 20 seconds.
4. Valve1 was closed, and the sensors resistance was given 60 seconds to reach a steady state. The classification result/sensors characteristic values appeared on the LCD.
5. The 4-neck chamber was disconnected from the fruit sample beaker, Valve1 was turned to fresh air, Valve2 was opened, the odor was pumped out, the chamber was aired out with fresh air for two minutes, before returning to Step 1 for the following operation.

**Figure 5.**
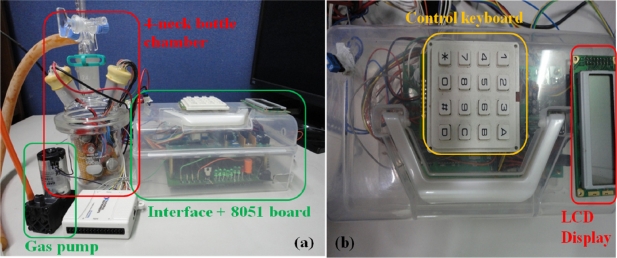
Pictures of experimental apparatus **(a)** 4-neck bottle chamber, gas pump, interface circuit, and the 8051 board; **(b)** control keyboard and LCD display.

The operational procedures are the same for the training and testing modes with the exception of Steps 1 and 4. For Step 1, set the 8051 to select operation mode. For Step 4, if the system is in training mode, sensor values are shown on the LCD; if the system is in testing mode, classification result of the target fruit is shown on the LCD. Figure 5 shows the apparatus used in the experiment. The sensors and the gas pump motor both fit into the transparent box (20 × 12 × 10 cm) which contains the interface circuitry and the 8051 board. The size, weight, and power dissipation of the different parts are shown in Table 2. Table 3 is a comparison between this work and some other portable electronic nose systems.

### 4.2. Experiment with the Odors of Three Fruits (Banana, Lemon, and Litchi)

**Figure 6.**
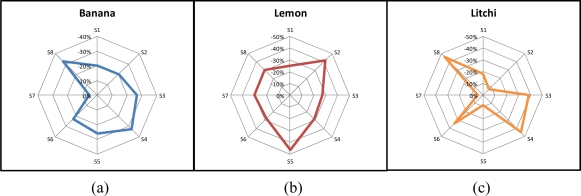
Fruit pattern of **(a)** banana, **(b)** lemon, and **(c)** litchi.

Three fruits (banana, lemon and litchi) were used to test the proposed E-Nose system. The data regarding the fruit odors was collected over a five-day span. On the first day, five different samples of each fruit were collected. The average response of the five samples was used as the odor signature for that fruit. Figure 6 shows the resulting patterns for testing the odor of the fruit samples. The magnitude of each axis indicates the resistance change ratio (ΔR/R) in each sensor when reaching equilibrium. A unique odor fingerprint of each of the three odors is shown in the figure. This is an indication of the potential to use non-specific sensor arrays to construct an odor database.

Between the second day and the fifth day, two series of experiments were conducted. In the morning, the fruit samples were purchased for that day (current day), and five different samples of each fruit were collected. In the afternoon, the fruit samples purchased on the first day were used, and five different samples of each fruit were collected. For the duration of the experiment, the temperature was 24–28 °C, the humidity was 59–78%, and the fruit samples weighed 8−15 grams. Table 4 is a summary of classification results for the six algorithms used in the verification software.

The result is shown as a fraction, whose denominator is the total number of samples, and the numerator is the number of samples correctly classified by the algorithm. For lemon and litchi, the total number of the current day samples was 19 and 18, respectively, due to data collection problems causing the sensors to not respond. Otherwise, the total number of samples would have been 20. In real applications, it may not be known which day the fruit is purchased, thus current day data and first day data were summed to provide the values in Table 5.

**Figure 7.**
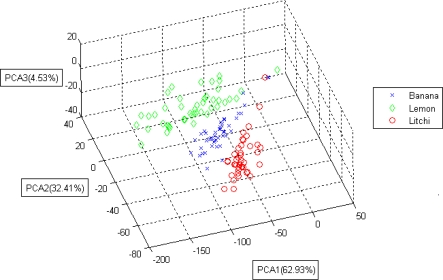
The PCA result of lemon, banana, and litchi.

Figure 7 shows a three-dimensional projection of the PCA results of all data points regarding the odor of the fruit.

From our experimental results, several inferences can be made:

1. The figure shows good recognition boundaries for the three fruit odors, and a high classification accuracy percentage was therefore expected.
2. A number of data points from the three classes were mixed; therefore, a certain degree of misclassification was expected.
3. Both the proposed portable E-Nose system (implemented with KNN) and the laptop verification software achieved an accuracy of 96.6% when identifying these three fruit odors.
4. The odor patterns of different fruits were distinguishable, enabling the possibility of recognizing the odor of fruit.
5. Although commercial gas sensors have specific target odors, they still respond to other gases (that are not stated in the datasheet as target odors), because of the sensing mechanism used. This is one of the main reasons for the interference problems of these sensors causing false alarms.
6. Even if specific commercial gas sensors are not designed for sensing the odor of fruits, with the help of proper recognition algorithms, effective fruit recognition systems could still be developed.

### 4.3. Experiments with Four Odors of Fruit (Banana, Lemon, Litchi, and Longan)

**Figure 8.**
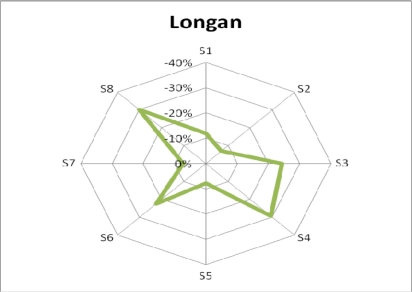
Fruit pattern of longan.

Banana, lemon, and litchi have very different odor fingerprints, because they have very different compositions, and very good classification accuracy is expected. In order to further validate the accuracy of the proposed E-Nose system, longan, a fruit that looks, smells, and tastes similar to litchi, was also tested in the experiments. Five samples of longan were collected on the first day, and the average response of these five samples was used as its corresponding odor signature. Figure 8 shows the resulting pattern of longan, which is very similar to that of litchi, as shown in Figure 6.

Between the second day and the fifth day, the same experiments were conducted on longan. Table 6 is a summary of the classification result for the six algorithms in the verification software for the four kinds of fruits. Table 7 shows the current day data and first day data summed together.

**Figure 9.**
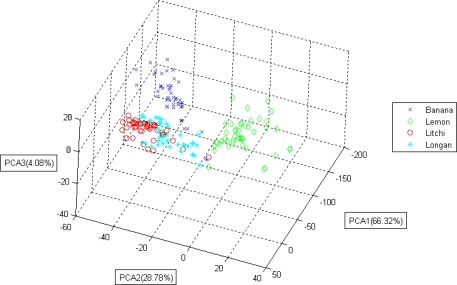
The PCA result of lemon, banana, litchi, and longan.

The overall classification accuracy in Table 7 (with the addition of longan) was lower than that in Table 5. This was expected, because the odor pattern of longan is very similar to that of litchi. The proposed E-Nose system implementing KNN algorithm was capable of 95% accuracy for banana and lemon, but only 70% accuracy between litchi and longan. This indicates that more sensors of different varieties may be needed in the future, to improve classification accuracy, particularly for odors of similar fruits. Figure 9 shows a three-dimensional projection of the PCA results for data points for each of the four kinds of fruit. It can be seen in the figure that a portion of the data points of litchi and longan fruit overlap.

## 5. Conclusions and Future Work

We have developed a prototype of a portable electronic nose comprising an interface PCB and a digital microprocessor board. We also developed and tested KNN classification algorithms. A parallel verification program was developed to verify the functions and the algorithms of the system. The prototype has been tested with three complex fruit odors, namely, lemon, banana, and litchi. The prototype of the proposed portable E-Nose system and the verification software achieved a classification accuracy in excess of 95%. This E-Nose prototype is highly suitable for implementation as a portable system.

## Figures and Tables

**Table 1.** The eight FIGARO^®^ sensors to form the sensor array.

**Sensor number**	**Sensor Type**	**Target gas (according to FIGARO^®^ datasheet)**
1	TGS2620	Alcohol, Solvent vapors
2, 5	TGS826	Ammonia
3, 6	TGS822	Alcohol, Solvent vapors
4, 8	TGS825	Hydrogen sulfide
7	TGS2602	General air contaminants

**Table 2.** Size, weight, and power dissipation of different parts of the E-Nose system.

**Part**	**Size**	**Weight**	**Power consumption**
4-neck bottle chamber	500 mL	886 g	8.88 W (sensor)
Interface and 8051 board	15 × 10 × 5 cm	627 g	0.96 W
Motor	9 × 5 × 5 cm	196 g	3.12 W
Total	20 × 12 × 10 cm	1,780 g	12.96 W

**Table 3.** Comparison of this work with other portable electronic nose systems.

	[17]	[18]	[19]	[25]	[39]	[40]	This work

No. Sensors	6	8	6	N/A	6	600	8
Target gas	26 carbon monoxide-hydrocarbon COrHC car exhausting gases	freshness of sardines	outdoor air monitoring of a duck breeding	hand-held electronic nose (H2EN)	recognition of flammable liquids	Oil/e-Mucosa System	Recognition of fruit odor

Sensor type	MOS	MOS (FIGARO)	MOS (FIGARO)	CP/MOS	MOS (FIGARO)	CP	MOS (FIGARO)
Size/weight	N/A	N/A	N/A	N/A	N/A	200 × 100 (mm)	200 × 120 × 100 (mm) /1,780g

Processor	Intel 80c196kc	PIC16F877/PC	Mitsubishi μ-controller (M16C)	PIC16F877/PC/PDA	PC	PIC18F8722/PIC18F4550	8051

**Table 4.** Summarized fruity odor classification result for the six algorithms.

		**SVM**	**PSVM**	**NN**	**PNN**	**KNN(K = 3)**	**PKNN(K = 3)**

Banana	Current day	15/20	20/20	18/20	19/20	18/20	18/20
	First day	15/20	20/20	20/20	20/20	20/20	20/20

Lemon	Current day	19/19	9/19	18/19	17/19	18/19	17/19
	First day	20/20	6/20	20/20	20/20	20/20	20/20

Litchi	Current day	16/18	17/18	17/18	18/18	17/18	18/18
	First day	15/20	16/20	20/20	18/20	20/20	19/20

**Table 5.** Total classification result for the three fruity odors.

	**SVM**	**PSVM**	**NN**	**PNN**	**KNN(K = 3)**	**PKNN(K = 3)**

Banana	30/40	40/40	38/40	39/40	38/40	38/40

Lemon	39/39	15/39	38/39	37/39	38/39	37/39

Litchi	31/38	33/38	37/38	36/38	37/38	37/38

Total	100/117	88/117	113/117	112/117	113/117	112/117
Accuracy	85.5%	75.2%	96.6%	95.7%	96.6%	95.7%

**Table 6.** Summarized fruity odor classification result for the six algorithms.

		**SVM**	**PSVM**	**NN**	**PNN**	**KNN(3)**	**PKNN**

Banana	Current day	18/20	20/20	18/20	19/20	18/20	18/20
	First day	20/20	20/20	20/20	18/20	20/20	19/20

Lemon	Current day	2/19	6/19	17/19	19/19	17/19	19/19
	First day	0/20	3/20	20/20	20/20	20/20	20/20

Litchi	Current day	15/18	13/18	9/18	17/18	6/18	16/18
	First day	12/20	18/20	12/20	19/20	12/20	19/20

Longan	Current day	5/17	5/17	13/17	10/17	15/17	12/17
	First day	4/19	15/19	18/19	5/19	18/19	6/19

**Table 7.** Total classification result for the three fruity odors.

	**SVM**	**PSVM**	**NN**	**PNN**	**KNN(3)**	**PKNN**

Banana (B)	38/40	40/40	38/40	37/40	38/40	37/40

Lemon (L1)	2/39	9/39	37/39	39/39	37/39	39/39

Litchi (L2)	27/38	31/38	21/38	36/38	18/38	35/38

Longan (L3)	9/36	20/36	31/36	15/36	33/36	18/36

Total	76/153	100/153	127/153	127/153	126/153	129/153
Accuracy (all)	49.7%	65.4%	83.0%	83.0%	82.4%	84.3%
Accuracy (B, L1)	50.6%	62.0%	94.9%	96.2%	94.9%	96.2%
Accuracy (L2, L3)	48.6%	68.9%	70.3%	68.9%	68.9%	71.6%
